# The global forum on bioethics in research meeting, “ethics of alternative clinical trial designs and methods in low- and middle-income country research”: emerging themes and outputs

**DOI:** 10.1186/s13063-019-3840-3

**Published:** 2019-12-19

**Authors:** Adrienne Hunt, Carla Saenz, Katherine Littler

**Affiliations:** 1 Wellcome Trust, 215 Euston Road, London, NW1 2BE UK; 2 Pan American Health Organisation, Washington, DC USA

**Keywords:** International research ethics; Global Forum on Bioethics in Research, Cluster randomised trials, Stepped wedge cluster randomised trials, Adaptive platforms, Controlled human infection models

## Abstract

Alternative clinical trial designs and methods are increasingly being used in place of the conventional individually randomised controlled trial (RCT) in high-income and in low-income and middle-income country (LMIC) research. These approaches - including adaptive, cluster-randomised and stepped-wedge designs and controlled human infection models - offer a number of potential advantages, including being more efficient and making the clinical trial process more socially acceptable. However, these designs and methods are generally not familiar to researchers, research ethics committees and regulators and their ethical implications have not received sufficient international attention from the bioethics, research, and policymaking communities working together. The ethics of alternative clinical trial designs and methods in LMIC research was chosen as a topic for the 2017 Global Forum on Bioethics in Research (GFBR). The meeting opened a global dialogue about this emerging issue in research ethics and gave voice to the LMIC perspective. It identified the need to take a multidisciplinary approach and to develop capacity amongst researchers and research ethics committees and regulators to propose, review and regulate these novel designs and methods. Building skills and infrastructure will empower researchers to choose from a broad range of designs and methods and adopt the most scientifically suitable, efficient, ethical and context-appropriate of these. The need for capacity development is most pressing from the LMIC perspective, where limited resources create an urgency to seek the most efficient trial design and method. The aim of this paper is to encourage broad debate about this complex area of research. By opening up this debate, GFBR aims to promote the appropriate and ethical use of novel designs and methods so their full potential to address the health needs in LMICs can be realised.

## Background

The Global Forum on Bioethics in Research (GFBR) was founded in 1999 as the principal global platform for debate on ethical issues pertaining to international health research [1]. Its core aims are to give voice to low-income and middle-income country (LMIC) perspectives as a priority in dialogue about global health research ethics, and to promote collaboration. The Forum plays a unique role, promoting international discussion of emerging global research issues and helps shape policy and practice. From 2015 it has convened annually, with each meeting centred on a key emerging theme of significance for global health research [2].

Alternative trial designs came to global attention during the Ebola outbreak in West Africa. Discussion arose as to whether randomised controlled trials (RCTs) - traditionally considered to be the most reliable way to generate evidence about an intervention - in an emergency context are ethical, feasible or have support of local communities [3]. Alternative trial designs - including adaptive, cluster randomised trials (CRTs) and stepped wedge (SW) designs - emerged in response [4], prompting discussion about their scientific value and ethical implications [5–7]. Similarly, the Zika virus outbreak brought renewed attention to alternative methods, such as controlled human infection models (CHIM), in response to the need to develop vaccines promptly.

Together, these alternative approaches can offer several potential advantages, including accelerating drug development, advancing a superior profile of benefits over risks and sometimes simplifying trial organization and field-work in low resource settings. However, their scientific merits for specific trials tend to be difficult to establish, and some of their ethical implications remain uncertain. Identifying when to opt for a new design or method, adequately calculating the study population size and relevant algorithms, comparing the efficiency of different possible approaches, establishing and weighing the risks and potential benefits to participants and selecting an adequate consent process are challenging. Current guidelines were largely written without special consideration of new trial designs and methods. Researchers, research ethics committees and regulators are thus in need of further guidance to help them plan, review and implement these often-complex trials.

**Fig. 1:**
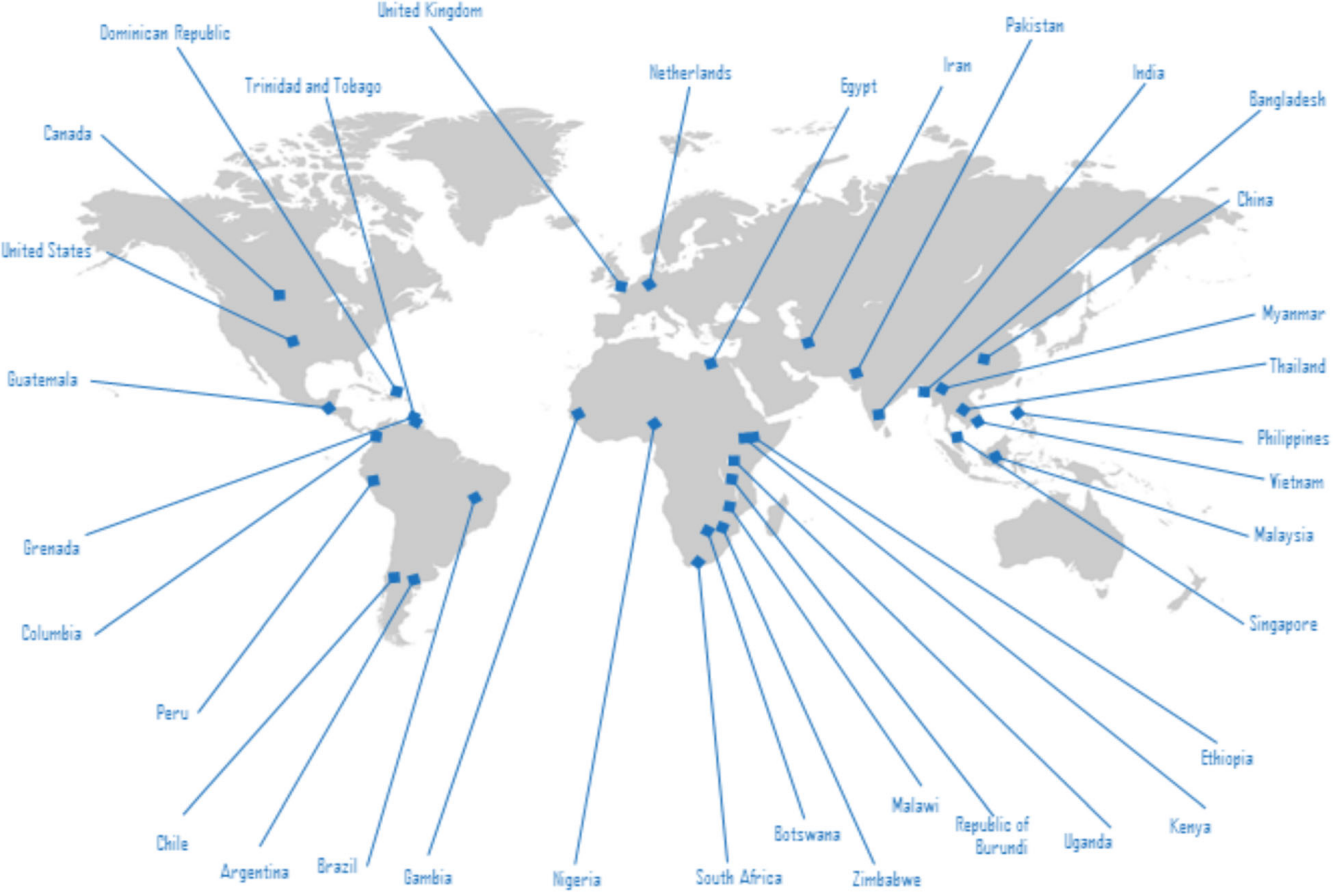
Global Forum on Bioethics in Research (GFBR) participants: 93 participants from 35 countries came together to discuss this important issue with a wide range of academic and clinical expertise: bioethicists, clinicians, statisticians, community practitioners, policymakers, social scientists, regulators and funders, at all levels of seniority. Of the participants, 58 were from low-income and middle-income countries (LMICs) Map taken from The Pixel/Shutterstock.com

The ethics of alternative clinical trial design and methods in LMICs research was therefore chosen as the topic for the 2017 GFBR meeting [8]. The meeting was held in Bangkok, Thailand over 2 days and brought together stakeholders from 35 countries (Fig. 1, Additional file 1), across fields of bioethics, clinical trials, statistics, epidemiology, public policy and clinical research. Using a case study format that enabled participants to understand the practical issues “on the ground” in addition to broader ethical and policy questions (Box 1), the meeting revealed complex issues and a diversity of opinion on the use of these designs and methods.

**Box 1:** GFBR meeting format

The case studies contained in this Supplement Issue formed the basis of the GFBR meeting and were themed by trial design and method: CRT, SW CRT, adaptive platform and CHIMs. Each session concluded with plenary discussion and was followed by focused small group discussion. The small groups comprised geographically diverse participants so each could learn from the others’ experience and point of view. Two panel sessions were dedicated to policy and guidance: The first session involved an overview of relevant international guidance and responses from a panel that offered regional perspective from East Africa, the Caribbean, Latin America and Southeast Asia. The second panel identified gaps in the guidance and proposed solutions. These included the use of simulation to address concerns about response-adaptive randomisation and to highlight the ethical advantages and disadvantages of the approach. GFBR participants also heard about a funder initiative to create guidance for CHIMs. *GFBR* Global Forum on Bioethics in Research, *CRT* cluster randomised trial, *SW* stepped wedge, *CHIM* Controlled human infection model	

## Main text

### Emerging themes

#### Choosing the best design or method for the research question

Novel designs and methods should not be labelled “alternative” as this suggests they are somehow flawed in comparison to the traditional individually RCTs. Instead we should ask what is the best design for the goals of the study. Indeed, it is not that one type of design or method is in general better or “more ethical” than others. The best designs and methods should be chosen based on the specific research question and the context in which the study is going to take place.

GFBR participants identified the following considerations as relevant to determining if non-traditional designs and methods are the most appropriate for a study:

- Scientific: validity, efficiency, objective of the study (e.g. if the goal is to measure or affect change at the community or population level).
- Ethical: risk minimization and maximization of benefits.
- Practical: recruitment, feasibility in implementation, costs, cultural or social preferences.

Having an understanding of the context where the research will take place is also important to determine which approach is ethically and/or scientifically preferable. The context will depend on a range of issues, including:

- Nature of health condition being studied (e.g. public/population health, emergency).
- Nature of available treatment (e.g. available range of therapeutic options, duration in evaluating study endpoints).
- Resources (e.g. availability of the intervention and personnel).
- Preference of stakeholders (e.g. community acceptability).
- Behaviour of participants (e.g. sharing of medicine).

The points above reflect discussion at the GFBR meeting in Bangkok and are not intended to be definitive. They are offered to prompt broader discussion on the range of relevant considerations.

#### Ethical oversight should be appropriate and flexible

Some of the novel designs - particularly SW trials - can be used to study how an intervention works and to evaluate the roll-out of a new intervention. Research and programme evaluation are however governed by different ethical and regulatory standards. The aims of the activity determine if it constitutes research or programme evaluation, and thus which requirements should be met, e.g. ethics review for research with human participants. Yet there are grey areas because the production of evidence is a continuous process. It may therefore be challenging to determine at what point the production of evidence that is necessary to justify rolling out an intervention has been completed, and at what point the transition to evaluation or quality improvement has been reached.

Ethical oversight should take these challenges into account. Moreover, it is important to separate out ethical and regulatory requirements to ensure that the ethical concerns are appropriately identified and scrutinised, instead of merely “ticking the boxes” or applying a checklist approach. In this context, it is important to ensure that a clear justification is provided for the approach taken (i.e. finding and justifying appropriate ethics input). With that goal in mind, the competencies to analyse the specific features of a novel research protocol should be strengthened, for which genuine partnerships between ethics and science throughout the research process seem crucial.

#### Local capacity should be developed to propose, review and regulate these designs and methods

There is limited international guidance on these trial designs and methods and scarce guidance specific to LMICs [9]. There is yet no consensus as to whether new ethical guidance for these designs and methods is needed, or whether LMIC-specific ethical guidance is needed. There is however consensus on the need to build capacity on the science behind the new designs, which is essential to assessing if the selection of the designs is justified and the risks and potential benefits that the designs or methods may pose to the research populations. There is further the worry that, in the absence of such understanding about these new trial designs and methods, countries will take a ‘precautionary’ approach, whereby research ethics committees (RECs) and regulators reject any kind of novel design or method.

International organizations such as the World Health Organization or the Pan American Health Organization (PAHO) can take the lead in developing these capacities, which include training in statistical calculations to determine sample sizes and the algorithms and mathematical models required to implement the designs. Training should be provided to researchers, RECs and regulators in how to develop and review these new designs and methods. Training could be reinforced by continuous engagement e.g. fora for researchers, ethicists, statisticians and regulators to meet regularly at the regional and local levels to raise awareness of the scientific reasons for employing these designs and methods and when they would be appropriate. In addition, funders could include additional provision for training/support of LMIC RECs, attached to specific projects, to enhance their confidence to assess novel trial designs and methods. Finally, if additional guidance for these designs and methods is deemed necessary, international organisations should take the lead issuing the guidance and ensuring regional representation in its development and advancing its uptake.

#### Community engagement is crucial for public acceptability of novel designs and methods

Public acceptability is crucial to the conduct of research. While the views of the communities in the locations where trials are being conducted must be respected, it should also be noted that communities may refuse to accept studies that are ethically sound. For example, some GFBR participants could not envisage CHIMs studies being accepted in their setting. Conversely, anecdotal evidence suggests that some communities may be more comfortable and thus likely to participate if they are all given the same intervention, that is, if there is no randomisation to different interventions. It is important to obtain empirical data on whether or not particular trial designs or methods are acceptable in practice to local communities and potential participants.

Underlying trust is crucial for public acceptability. Public acceptability and trust arose in particular in the context of CHIM studies, where volunteers are deliberately infected with a well-characterised strain of an infectious agent. It is important to ensure participants understand the associated risks, which will depend on how well the disease characteristics are understood (e.g. malaria versus Zika). One case study described research participants who were willing to be infected with malaria, which was endemic in their region, as they had a daily life comparator and understood malaria is curable.

Community engagement contributes to building trust in research and is vital for addressing the beliefs or cultural norms that may impact on public acceptability of novel designs and methods. Public acceptability is susceptible to change based on whether the relevant information has been adequately provided, and how that information was delivered. Community engagement strategies should be informed by empirical research with communities and regulators to find out what is important to them, including preferences for how information is provided.

#### Researchers and sponsors should involve government and other stakeholders in advance for trials that are meant to impact policy

For research to have the greatest impact, governments and policy makers must have confidence in the validity of the results and be willing to act on the findings. Otherwise, this calls into question the social value in conducting trials if there will be no uptake. Engagement with appropriate level(s) of government (sub-national, district, etc.) and policy-makers should start in advance of the trial and address both implementation and sustainability. This is especially important in contexts where non-traditional designs are unfamiliar, and where the resulting evidence may be seen as inferior to evidence from a traditional RCT. For example, if CRTs are not recognised as “gold standard” evidence, this may affect the way in which the evidence they produce is taken up in practice.

#### Meeting outputs

GFBR participants are selected competitively, based on their potential to actively contribute to the discussions and to achieve impact after the meeting. Participants are encouraged to report the meeting recommendations in their home countries and to continue the discussion in their local context. GFBR participants have given presentations on the meeting theme to their local RECs and at other conferences and in published papers [10]. Materials from the meeting have been incorporated into university course materials around the globe.

One of the most significant outputs to date came from a group of Latin American participants, including ethicists, researchers, ethics committee members and representatives of health authorities from USA, Argentina, Brazil, Chile, Colombia, Guatemala, Peru and Dominican Republic. The group published a paper in the PAHO’s Public Health Journal, which is widely read in the region and thus highly influential [11]. The authors argue for the ethical duty of all parties involved in research for health to choose from *all* possible methods and designs - as opposed to only from those with which we are familiar - to ensure that clinical trials carried out in Latin America have the most scientifically suitable designs and methods to answer the research question efficiently, and that research is carried out according to the highest ethical standards. A determined effort to update the relevant regional capacities is essential to achieve this. This output exemplifies the key aims of the GFBR to promote global dialogue on ethical issues, to enable new networks and collaborations to form across disciplines and countries and to inspire GFBR participants to take up the issue and address the challenges in their local context.

#### Fellowship scheme

Forum participants are encouraged to develop collaborations and new networks resulting from the meeting, which may be supported through the GFBR fellowship scheme. The scheme was launched after the 2015 meeting and has supported 18 Fellows from 15 countries. The scheme provides an opportunity for GFBR participants to explore issues that arise during a GFBR meeting in greater detail, establish new collaborations, and develop new ideas for resolving issues that could not be resolved at the meeting itself. The scheme is geared towards encouraging north/south and south/south collaboration and is open to LMIC-based colleagues. So far, Fellows have produced a range of outputs including peer-reviewed papers, regional workshops on the meeting theme, conference presentations, country guidance and online educational resources.

After the 2017 meeting, four fellowships were awarded through a competitive process. The GFBR Fellows come from Colombia, Malawi, India and the Philippines and their collaborators are drawn from Thailand, UK, Canada, USA, Brazil and Argentina. The Fellows will host regional meetings on the GFBR topic, for REC members and others, develop country specific guidance for CHIMs and assess the ethical issues involved in HIV implementation research that have used CRT design and SW trial design.

The fellowships not only promote new collaborations but ensure the legacy of the 2017 GFBR meeting by giving rise to outputs that further encourage dissemination and discussion about the ethics of novel designs and methods.

## Conclusions

Novel trial designs and methods can offer a number of potential advantages over the conventional individually randomised clinical trial. They enrich a researcher’s armoury, allowing them to choose from a broader range of designs and methods and to adopt the most scientifically suitable, efficient, ethical and context-appropriate of these. However, there is still a long way to go until researchers in LMICs can effectively resort to them. Moreover, ethical implications of these alternative approaches have not received sufficient international attention.

This GFBR meeting brought together the global bioethics and research community and regulators to discuss this emerging issue in research ethics. During the discussion it became clear that novel designs and methods are largely unfamiliar to the research community and there is limited international guidance to support their implementation. In this context, researchers may limit themselves - or be limited by ethics review processes or regulation - to only the subset of clinical trials with which they are familiar, rather than selecting the most efficient. This global issue is most pressing from the LMIC perspective, where limited resources create an urgency to seek the most efficient trial design and method.

Ideally, novel designs and methods should be given equal consideration to traditional models. However, the required infrastructure is lacking, especially in LMICs. Capacity development is urgently required to build the necessary skills to consider, review and implement these designs and methods. The complexity of the issue highlights the need for science and ethics to work together and for a multidisciplinary approach to doing research ethically in resource constrained settings. Critically, the ethics discourse requires input from trialists and statisticians.

The GFBR provided a mutual ground for discussion and a shared understanding of the challenges and opportunities presented by novel designs and methods. It opened up a global dialogue, shedding light on the outstanding questions, such as knowing what effective community engagement looks like for these complex trials. Many GFBR participants have continued the meeting discussion in their regional and national contexts. The aim of this paper is to draw attention to the issues and prompt discussion in the wider community. By opening up this debate, GFBR aims to promote the appropriate and ethical use of novel designs and methods so their full potential to address the health needs in LMICs can be realised.

## Supplementary information

**Additional file 1: Figure 1.** GFBR participants

## Data Availability

Not applicable.

## Abbreviations

CHIMs: Controlled human infection models
CIOMS: Council of International Organizations of Medical Sciences
CRTs: Cluster randomised trials
GFBR: Global Forum on Bioethics in Research
LMIC: Low-income and middle-income country
PAHO: Pan American Health Organization
RCT: Randomised controlled trial
REC: Research ethics committee
SW: Stepped wedge
