# Supplementary material for: The global forum on bioethics in research meeting, “ethics of alternative clinical trial designs and methods in low- and middle-income country research”: emerging themes and outputs
Source: Trials. 2019 Dec 19;20(Suppl 2):701. doi: 10.1186/s13063-019-3840-3 (PMC6921436; doi:10.1186/s13063-019-3840-3)
Supplement: Supplementary file 1 — Additional file 1: Figure 1. GFBR participants [file 13063_2019_3840_MOESM1_ESM.docx]

**Reviewer’s Report**

**Article Title: The global forum on bioethics in research meeting, “ethics of alternative clinical trial designs and methods in low- and middle-income country research”: emerging themes and outputs**

**Date: 22/03/2019**

**Reviewer name:** Karla Hemming

**Reviewer’s report:**

Overall, as a meeting report, I think it is a very good summary.

As a meeting report, this does not quite fit a conventional review but I’ve some comments:

“Alternative clinical trial designs and methods are increasingly being used in place …”

*I think the focus is on new trial designs and it is unclear to me exactly what is meant by “methods”*

“However, these designs and methods are generally not familiar to researchers, research ethics committees and regulators, and their ethical implications have not received sufficient international attention from the bioethics, research, and policymaking communities working together…”

*This is one good motivation for considering the ethics of these trials designs. But, I don’t think the case is made for considering this in LMICs.*

**Level of interest. Please indicate how interesting you found the manuscript:**

High

**Quality of written English. Please indicate the quality of language in the manuscript:**

Good

**Quality of figures; All images and figures within the manuscript should be genuine i.e. without evidence of manipulation. No specific feature within an image may be enhanced, obscured, moved, removed, or introduced. If you have concerns about the veracity of the figure, please comment below.**

n/a

**Statistical review**

Is it essential that this manuscript is seen by an expert statistician? If so, please give your reasons in your report above.

no

**Declaration of competing interests**

Please complete a declaration of competing interests, considering the following questions:

1. Have you in the past five years received reimbursements, fees, funding, or salary from an organisation that may in any way gain or lose financially from the publication of this manuscript, either now or in the future?

2. Do you hold any stocks or shares in an organisation that may in any way gain or lose financially from the publication of this manuscript, either now or in the future?

3. Do you hold or are you currently applying for any patents relating to the content of the manuscript?

4. Have you received reimbursements, fees, funding, or salary from an organization that holds or has applied for patents relating to the content of the manuscript?

5. Do you have any other financial competing interests?

6. Do you have any non-financial competing interests in relation to this paper?

If you can answer no to all of the above, write 'I declare that I have no competing interests' below. If your reply is yes to any, please give details below.

I declare that I have no competing interests

**Open Peer Review Declaration**

I agree to the open peer review policy of the journal. I understand that my name will be included on my report to the authors and, if the manuscript is accepted for publication, my named report including any attachments. I upload will be posted on the website along with the authors' responses. I agree for my report to be made available under an Open Access Creative Commons CC-BY license (http://creativecommons.org/licenses/by/4.0/). I understand that any comments which I do not wish to be included in my named report can be included as confidential comments to the editors, which will not be published.

Karla Hemming

**Were you mentored through this peer review?**

No

**Reviewer’s Report**

**Article Title: The global forum on bioethics in research meeting, “ethics of alternative clinical trial designs and methods in low- and middle-income country research”: emerging themes and outputs**

**Date:** March 15, 2019

**Reviewer name:** Charles Weijer

**Reviewer’s report:**

This is a well written and accurate report on the Global Forum for Bioethics in Research meeting in Bangkok in 2017. The authors have described the motivation and goals for the meeting clearly. Further, they have done an excellent job of distilling a vibrant 2-day discussion into overarching themes. I have no suggestions for improvement.

**Level of interest. Please indicate how interesting you found the manuscript:**

High.

**Quality of written English. Please indicate the quality of language in the manuscript:**

Excellent.

**Quality of figures; All images and figures within the manuscript should be genuine i.e. without evidence of manipulation. No specific feature within an image may be enhanced, obscured, moved, removed, or introduced. If you have concerns about the veracity of the figure, please comment below.**

Not applicable.

**Statistical review**

Is it essential that this manuscript is seen by an expert statistician? If so, please give your reasons in your report above.

No.

**Declaration of competing interests**

Please complete a declaration of competing interests, considering the following questions:

1. Have you in the past five years received reimbursements, fees, funding, or salary from an organisation that may in any way gain or lose financially from the publication of this manuscript, either now or in the future?

2. Do you hold any stocks or shares in an organisation that may in any way gain or lose financially from the publication of this manuscript, either now or in the future?

3. Do you hold or are you currently applying for any patents relating to the content of the manuscript?

4. Have you received reimbursements, fees, funding, or salary from an organization that holds or has applied for patents relating to the content of the manuscript?

5. Do you have any other financial competing interests?

6. Do you have any non-financial competing interests in relation to this paper?

If you can answer no to all of the above, write 'I declare that I have no competing interests' below. If your reply is yes to any, please give details below.

I was a member of the planning committee for the GFBR 2017 meeting, and I both attended and spoke at the meeting.

**Open Peer Review Declaration**

I agree to the open peer review policy of the journal. I understand that my name will be included on my report to the authors and, if the manuscript is accepted for publication, my named report including any attachments. I upload will be posted on the website along with the authors' responses. I agree for my report to be made available under an Open Access Creative Commons CC-BY license (http://creativecommons.org/licenses/by/4.0/). I understand that any comments which I do not wish to be included in my named report can be included as confidential comments to the editors, which will not be published.

Charles Weijer

**Were you mentored through this peer review?**

No

**Authors’ responses to reviewers: The global forum on bioethics in research meeting, “ethics of alternative clinical trial designs and methods in low- and middle-income country research”: emerging themes and outputs**

**Date:** 03 April 2019

Thank you for providing the reviewer reports. We note there are no comments from Charles Weijer and only two comments from Karla Hemming that relate to the abstract, and which do get addressed in the manuscript itself. We offer the following response but on balance do not consider that the comments call for changes to the abstract:

• I think the focus is on new trial designs and it is unclear to me exactly what is meant by “methods”

In the context of this meeting, "method" refers to Controlled Human Infection Models (CHIMs). CHIMs are not a design in the same sense that you might assess an investigational product by designing a 'cluster' trial, a 'stepped wedge' trial or an 'adaptive' trial. CHIMs is a distinct method involving healthy adult volunteers being infected with a well characterised strain of an infectious agent in highly controlled conditions to assess the mechanisms and determinants of immunity.

• This is one good motivation for considering the ethics of these trials designs. But, I don’t think the case is made for considering this in LMICs.

The GFBR exists to provide a global platform for the exchange and sharing of experience and expertise on research ethics and, most especially, it aims to amplify the voice of lower and middle income countries (LMIC) in these discussions. If the case has been made for considering the ethics of alternative designs and methods then, by default, this should be inclusive of both High Income Countries (HIC) and LMIC perspectives.

It is clear from the meeting that there are many challenges regarding the implementation and review of these designs and methods. If these challenges are hard in terms of relevant capacities in HICs, then we can be sure they are even more challenging in LMICs. As argued in the abstract and paper, the need for capacity development is most pressing from the LMIC perspective, where limited resources create an urgency to seek the most efficient trial design and method. It is for this reason that GFBR decided to focus on this topic to encourage broad debate about this complex area of research.

With thanks and best wishes,

Adrienne, Carla and Katherine
